# Colitis nucleomigrans: The third type of microscopic colitis (part 2). An ultrastructural study

**DOI:** 10.1111/pin.12995

**Published:** 2020-08-06

**Authors:** Mitsuhiro Tachibana, Yutaka Tsutsumi

**Affiliations:** ^1^ Departments of Diagnostic Pathology Shimada Municipal Hospital Shizuoka Japan; ^2^ Departments of Diagnostic Pathology Clinic Pathos Tsutsumi Aichi Japan

**Keywords:** apoptosis, colitis nucleomigrans, electron microscopy, microscopic colitis

## Abstract

In the preceding article (part 1), we proposed the third type of microscopic colitis: colitis nucleomigrans (CN). Microscopically, the nuclei of surface‐lining columnar cells were migrated in chain to the middle part of the cells, and apoptotic nuclear debris was scattered in the cytoplasm beneath the nuclei. For ultrastructural analysis, buffered formalin‐fixed biopsy tissue of CN (*n* = 2) was dug out of paraffin blocks. After deparaffinization, tissue blocks were prepared with conventional sequences. Ultrathin sections were stained with uranyl acetate and lead citrate. Fine morphological preservation was satisfactory even after paraffin embedding. Apoptotic nuclear debris was localized within the cytoplasm beneath the migrated nuclei of the surface‐lining columnar cells. Abnormality of cytoskeletal filaments (actin, cytokeratin and tubulin) was scarcely recognized in the epithelial cytoplasm. Macrophages located in the uppermost part of the lamina propria phagocytized electron‐dense globular materials. Intraepithelial lymphocytes with scattered dense bodies were observed among the columnar cells. We suppose that altered apoptotic processes in the colorectal surface‐lining epithelial cells may be involved in the pathogenesis of CN. Mechanisms of nuclear migration to the unusual position or impairment of nuclear anchoring to the basal situation in the surface‐lining epithelial cells remain unsettled, because cytoskeletal components showed little ultrastructural abnormality.

**Abbreviations**
CN: colitis nucleomigrans
IBD: inflammatory bowel disease
MC: microscopic colitis

## INTRODUCTION

Ultrastructural characteristics of apoptosis have been well described microscopically during the last four decades.^1^, ^2^, ^3^ The initial stage of apoptosis is characterized by heterochromatin margination along the nuclear membrane but with intact cytoplasmic organelles. Eventually, apoptotic nuclear debris is phagocytized by macrophages as apoptotic bodies. Apoptosis occurs not only in normal cells and tissues physiologically but also under abnormal/diseased conditions.^4^ Activation (cleavage) of endogenous endonucleases, particularly caspase‐3, induces apoptotic cell death, through a process of DNA degradation to inter‐nucleosomal fragments of a 180 base pair unit and multiples thereof.^5^

In the preceding article (part 1),^6^ we proposed the third type of microscopic colitis (MC), named colitis nucleomigrans (CN). We describe herein ultrastructural features of the CN by using endoscopic biopsy samples fixed in neutral‐buffered 10% formalin and embedded in paraffin. As was described previously,^7^ the usefulness of routinely prepared paraffin blocks for ultrastructural observation should be emphasized.

## MATERIALS AND METHODS

### Patients

Among 33 cases analyzed in the partner article (part 1),^6^ two biopsy lesions from a 54‐year‐old male and a 70‐year‐old male were chosen for electron microscopic study. Both cases suffered from CN with inflammatory bowel disease (IBD)‐like symptoms.

### Ultrastructural analysis

Routinely prepared paraffin blocks were processed for ultrastructural observation, as reported previously.^7^ Colonoscopic biopsy samples of CN were fixed in phosphate‐buffered 10% formalin, pH 7.4 (Kanto Chemical, Tokyo, Japan), and embedded in paraffin (Parabett 60 GR, Muto Pure Chemicals, Tokyo, Japan). The biopsy pieces were dug out of the paraffin blocks of the two cases of CN as small cubes using a single edge industrial blade. After deparaffinization overnight, the tissue blocks were dehydrated in graded series of alcohol, re‐fixed at 4°C overnight in 2.5% glutaraldehyde (Yuai Kasei, Amagasaki, Japan) buffered with 0.1 M sodium cacodylate at pH 7.4, osmified for 2 h with sodium cacodylate‐buffered 1% osmium tetraoxide (Nisshin EM, Tokyo, Japan), embedded in epoxy resin (Epok 812, Okenshoji, Tokyo), and polymerized overnight in a 70°C oven. Ultrathin sections were cut using a Diatome diamond (JEOL Japan, Tokyo, Japan) at 80 nm thickness, and stained with uranyl acetate (Ieda Chemicals, Tokyo, Japan) and lead citrate (Sigma Aldrich Japan, Tokyo, Japan). Images were photographed on a JEOL JEM1400Flash Electron microscope (JEOL Japan) equipped with an EM‐14661FLASH high‐sensitivity digital complementary metal‐oxide‐semiconductor camera.

### Ethics approval

All the procedures were in accordance with the ethical standards of the hospital committee on human experimentation and with the 1964 Helsinki Declaration and later versions. Written informed consent was obtained from both of the patients after selection of cases. The study was approved in December 2019 by the Ethics Committee for Clinical Research of Shimada Municipal Hospital, Shimada, Shizuoka (approval number R01–10).

## RESULTS

**Figure 1:**
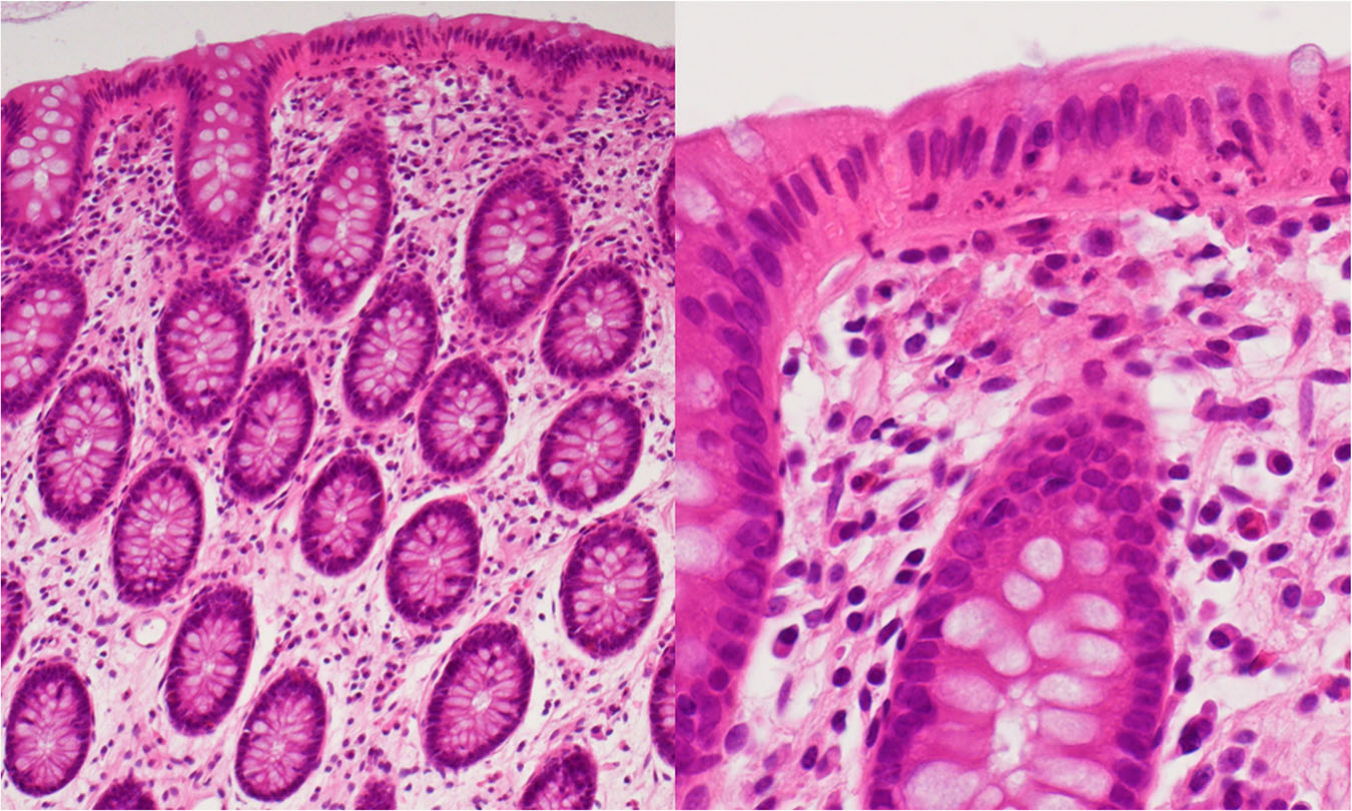
Light microscopic findings of colitis nucleomigrans (CN) with inflammatory bowel disease (IBD)‐like symptoms (54 years‐old male; hematoxylin and eosin (HE); left, low‐powered view; right, high‐powered view). The nuclei of the surface‐lining columnar cells are migrated in chain to the middle part of the cells. Eosinophilic cytoplasm is evident beneath the chained nuclei, and apoptotic bodies (clustered fragments of nuclear debris) are observed in the cytoplasm. Nuclear migration is not observed in the colonic crypt. The lamina propria mucosae reveals moderate lymphoplasmacytic infiltration and superficial accumulation of macrophages. Neither subepithelial collagen bands nor increased intraepithelial lymphocytes (IELs) are noted.

Hematoxylin and eosin stained sections of the biopsy specimen revealed typical light microscopic appearance of CN (Fig. 1). Both light and electron microscopic findings were comparable in both lesions of CN evaluated. Ultrastructurally, the nuclei of the surface‐lining columnar cells were migrated in chain to the middle part of the cells, while the nuclei of colorectal crypts were anchored adjacent to the basement membrane. Apoptotic nuclear debris with varied electron density was dispersed in the infranuclear cytoplasm of the columnar cells. Macrophages distributed in the uppermost part of the lamina propria mucosae actively phagocytized electron‐dense globular materials. Capillary vessels, lymphocytes and plasma cells were also seen in the lamina propria. Intraepithelial lymphocytes (IELs) were observed among the columnar cells and near the basement membrane. The cytoplasm of IELs occasionally contained small‐sized and rounded dense bodies: the diameter of the granules ranged from 200 to 380 nm (mean 315). Features of translocation of the distorted nucleus through the basement membrane toward the lamina propria were also encountered.

The cytoplasm of surface‐lining columnar cells was rich in mitochondria. No abnormal aggregation or reorganization of fine (actin), intermediate‐sized (cytokeratin) and microtubular (tubulin) filaments was recognized.

**Figure 2:**
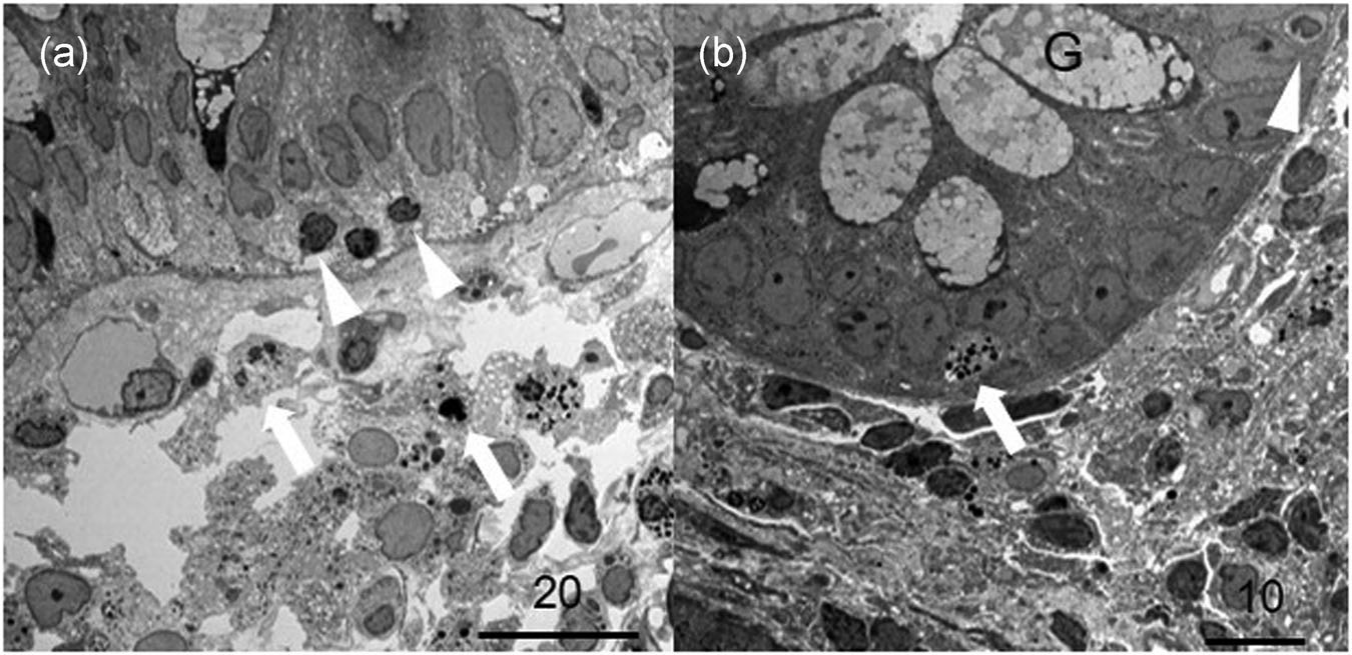
Low‐powered views of transmission electron microscopy for colitis nucleomigrans (CN) with inflammatory bowel disease (IBD)‐like symptoms. (**a**) The nuclei of the surface‐lining columnar cells are migrated in chain to the middle part of the cells. Intraepithelial lymphocytes (IELs) are observed beneath the migrated nuclei of the columnar cells (arrowheads). In the uppermost part of the lamina propria mucosae, macrophages phagocytizing electron‐dense apoptotic bodies (arrows) are clustered, and capillary vessels and lymphocytes are also dispersed (Bar = 20 μm). (**b**) In the crypt, the nuclei are uniformly located at the base of the columnar cells. G indicates goblet cells. An IEL is indicated by an arrowhead, and apoptotic debris (arrow) is seen among the epithelial cells (Bar = 10 μm).

**Figure 3:**
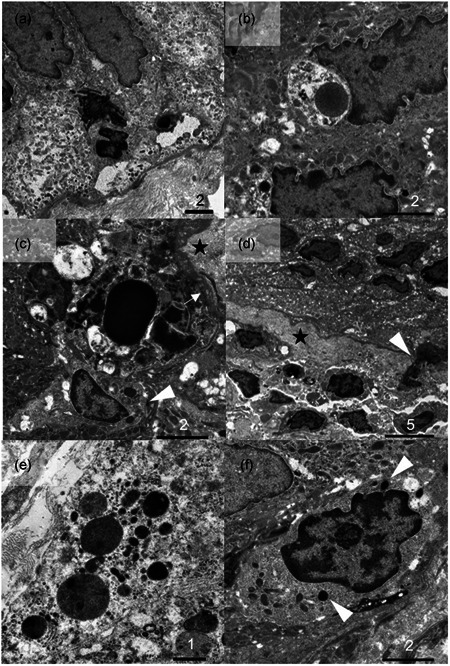
Higher‐powered views of transmission electron microscopy for colitis nucleomigrans (CN) with inflammatory bowel disease (IBD)‐like symptoms. (**a**) Two apoptotic and distorted nuclei appear to be located within the cytoplasm of the columnar cells rich in mitochondria. No aggregation or reorganization of cytoskeletal filaments is observed in the infranuclear cytoplasm (Bar = 2 μm). (**b**) Another field demonstrates an apoptotic debris engulfed by the enterocyte. The apoptotic debris is seen in the Golgi area of the columnar cells (Bar = 2 μm). (**c**) Electron‐dense apoptotic debris is distributed above the basement membrane (asterisk) and adjacent to an intraepithelial lymphocyte (IEL) (arrowhead). As indicated by an arrow, the cytoplasm of the columnar cells is discernible above the basement membrane (Bar = 2 μm). (**d**) A distorted nucleus (arrowhead) translocates through the basement membrane (asterisk) toward the lamina propria mucosae. The enterocytic cytoplasm beneath the migrated nuclei is rich in mitochondria without abnormality of cytoskeletal filaments (Bar = 5 μm). (**e**) A close view of a macrophage seen in the uppermost part of the lamina propria, actively phagocytizing electron‐dense apoptotic nuclear debris (Bar = 1 μm). (**f**) Representative appearance of an IEL situating among the surface‐lining columnar cells. As arrowheads indicate, round‐shaped and small‐sized dense bodies (mean diameter 315 nm) are dispersed in the cytoplasm of the IEL (Bar = 2 μm).

Figures 2 and 3 illustrate representative ultrastructural findings.

## DISCUSSION

Apoptosis in the intestinal epithelium is widely recognized as an important process for the maintenance of homeostasis balancing between cell proliferation and cell death. Apoptosis is distinct from necrosis in that it is a programmed form of cell death and occurs without accompanying inflammation. Activation (cleavage) of endogenous endonucleases, particularly caspase‐3, induces apoptotic cell death, through a process of DNA degradation to inter‐nucleosomal fragments of a 180 base pair unit and multiples thereof.^5^ Apoptosis is seen not only under normal (physiological) conditions but also in abnormal (pathological) states of cells and tissues.^4^ Abnormality of enterocytic apoptosis has been linked to a variety of intestinal disorders.^8^, ^9^

Chained nuclear migration to the middle part of the surface‐lining epithelial cells and accumulation of apoptotic nuclear debris beneath the migrated nuclei were unique and pathognomonic of CN and easily recognizable in hematoxylin and eosin stained biopsy specimens, as shown in Fig. 1.^6^ Such microscopic findings of CN were scarcely observed in collagenous colitis and the control normal colorectal mucosa.^6^ CN was subdivided to two categories: MC‐like type (accompanying watery diarrhea and normal endoscopic appearance) and IBD‐like type (accompanying occult or gross hematochezia and endoscopic mucosal reddening). Apoptotic nuclear debris in the diseased mucosa was more easily observed in the IBD‐like type than in the MC‐like type.^6^

The present ultrastructural analysis was performed in order to confirm the light microscpic features of CN. Our purpose of study was achieved by analyzing two cases, we believe. Apoptotic nuclear debris was seen within the infranuclear cytoplasm of the surface‐lining epithelial cells, and some of the distorted nuclei showed translocation through the basement membrane toward the lamina propria mucosae. Sträter *et al*.^10^ reported two modes of enterocytic apoptosis in normal human colonic mucosa: apoptotic bodies (debris) were engulfed by adjacent epithelial cells or apoptotic cells with only subtle morphological changes extruded into the gut lumen. The engulfment pattern was seen predominantly in the crypt, while the extrusion pattern was restricted to the luminal mucosal surface. In colonic adenomas, both patterns were accelerated but with predominance of the extrusion pattern. In CN, engulfment‐type apoptosis of the surface‐lining cells was significantly accelerated: an altered mode of apoptosis (switching from the extrusion type to debris type) seems to be characteristic of the diseased colorectal mucosa of CN. Regarding the fate of the apoptotic nuclear debris, Iwanaga *et al*.^11^ reported unique features of apoptosis at the tip of small intestinal villi of the guinea pig. The apical cytoplasmic plates without nuclei were pinched off into the lumen, and the nuclei were phagocytized by macrophages in the uppermost part of the lamina propria mucosae. It seemed that in CN, CD68‐positive macrophages located just beneath the basement membrane were activated to phagocytize apoptotic nuclear debris of enterocyte origin.

Generally speaking, the engulfment‐type apoptosis or formation of ‘apoptotic bodies’ represents common and typical microscopic appearance of programmed cell death.^12^ Apoptotic bodies seen in epithelial tissue are engulfed by adjacent epithelial cells, as was so in the surface‐columnar cells of the colon in CN. When apoptosis occurs in the stroma or apoptotic epithelial cells are sent to the stroma, macrophages actively phagocytize the apoptotic bodies, as seen in the lamina propria mucosae in CN. It is of note that the exclusion‐type apoptosis is a unique form seen in the gastrointestinal mucosa. The apoptotic cells distributed in the surface epithelial layer are regularly thrown away into the gut lumen as a ‘rounded’ apoptotic nuclei.

The chained nuclear migration to the middle part of the surface‐lining columnar cells should be the unique event in CN.^6^ It is hypothesized that abnormality of cytoskeletal proteins may impair localization of the nuclei that are normally anchored to the basal part of the columnar cells. Wang *et al*.^13^ analyzed the molecular basis for the regulation of apoptosis in the gastrointestinal epithelium and proposed cell biological mechanisms that couple changes in actin dynamics to apoptotic cell death. However, our current study failed to identify significant fine morphological changes of intracellular filament systems, including actin microfilaments, cytokeratin intermediate filaments and microtubules composed of tubulin proteins, in the enterocytic cytoplasm beneath the migrated chained nuclei. Further molecular analyses are necessary to settle the mechanisms and implications of chained nuclear migration in CN.

Intraepithelial lymphocytes of CD8‐positive cytotoxic T‐cell type are known to promote epithelial apoptosis by secretion of cytotoxic molecules such as granzymes and perforin: granzyme B provokes apoptosis through both caspase‐dependent and ‐independent pathways.^14^ In the present study, IELs possessed dense bodies, candidate granules containing the cytotoxic substances. IELs play key roles in a variety of intestinal disorders, including cryptal apoptosis and ulceration in ulcerative colitis,^15^ mucosal damage and epithelial apoptosis in celiac disease,^16^, ^17^ and accelerated epithelial apoptosis in human immunodeficiency virus infection.^18^ Changes of the microbiotas in the gut lumen may influence apoptosis of colorectal epithelial cells.^19^, ^20^, ^21^ In the preceding article (part 1),^6^ we showed that cleaved caspase‐3‐immunoreactive apoptotic nuclear debris was more frequently observed in CN with IBD‐like symptoms than in CN with MC‐like symptoms, while the number of CB8‐positive IELs was comparable and not significantly increased as seen in lymphocytic colitis, a form of MC. Supposedly, accelerated apoptosis of debris type may provoke occult/gross hematochezia, representing IBD‐like features.

Finally, we would like to emphasize the usefulness of routinely prepared paraffin blocks for the ultrastructural observation.^7^ If sections are cut from the paraffin blocks and mounted onto glass slides, the fine morphology is very poor. However, when the target lesions are dug out of the paraffin blocks using a blade as small cubes, the ultrastructural morphology should be satisfactory enough, as presented in the present article.

Further clinicopathological, ultrastructural, and molecular biological studies are needed in order to clarify unanswered questions in the perplexing condition, including the cause, pathophysiology, molecular mechanism and optimal treatment of CN.

## DISCLOSURE STATEMENT

None declared.

## AUTHOR CONTRIBUTIONS

Both authors have participated sufficiently in the work to take public responsibility for appropriate portions of the content: MT and YT cooperatively designed the study, analyzed both light and electron microscopic features, and approved the final manuscript. YT proposed a basic idea of CN.
